# Intelligent-assistant support versus usual self-scheduling for extracurricular physical activity among university students: a comparative evaluation

**DOI:** 10.3389/fpubh.2026.1945553

**Published:** 2026-10-07

**Authors:** Xu Li, Yuhao Jiang, Yuping Wang, Yingtao Wang, Yongfeng Liu

**Affiliations:** 1 School of Physical Education, Xi Jinping Collaborative Innovation Team for Research and Interpretation of Important Discourses on Cyberspace Governance, Chongqing University of Posts and Telecommunications, Chongqing, China; 2 School of Sports Training, Chengdu Sport University, Chengdu, Sichuan Province, China; 3 School of Life Health Information Science and Engineering, Chongqing University of Posts and Telecommunications, Chongqing, China; 4 School of Foreign Language and Business (School of International Education), Lianyungang Normal College, Lianyungang, China

**Keywords:** behavioural support, digital health promotion, extracurricular physical activity, intelligent assistant, university students, young adults

## Abstract

**Background:**

Universities require scalable approaches to promote physical activity among young adults. Digital support may influence not only how often students participate in physical activity, but also how their participation is distributed across different activity types. This study examined whether intelligent-assistant support integrating timetable-informed scheduling, adaptively timed reminders based on evolving exercise habits, and immediate feedback was associated with extracurricular physical activity patterns and end-of-semester anthropometric and fitness outcomes, compared with usual self-scheduling.

**Methods:**

This semester-long non-randomised comparative study included 6,530 undergraduates, of whom 3,258 used intelligent-assistant support and 3,272 followed usual self-scheduling. The two approaches operated within existing physical education course sections. Both groups were subject to the same institutional requirement of 45 extracurricular physical activity sessions. The main physical activity measures were total activity sessions, the proportion of sessions involving running, and mean distance per running session. Additional measures were body mass index, vital capacity, and sex-specific endurance-run time. Between-group differences were examined using Mann–Whitney U tests and covariate-adjusted linear regression.

**Results:**

Compared with usual self-scheduling, intelligent-assistant support was associated with 0.58 fewer total activity sessions, a 2.13-percentage-point greater proportion of sessions involving running, and a 0.061-km greater mean distance per running session. Vital capacity was 41.4 mL higher, and endurance-run time was 2.68 s lower among male students, whereas no between-group difference was observed among female students. Body mass index was 0.36 kg/m2 higher.

**Conclusion:**

Under a common activity target and monitoring environment, intelligent-assistant support was associated with small shifts in the composition of extracurricular physical activity, particularly towards running, but not with greater overall participation or consistently more favourable anthropometric and fitness outcomes.

## 1 Introduction

Regular physical activity is a major determinant of population health, contributing to cardiometabolic health, functional capacity, psychological well-being and the prevention of non-communicable diseases (1). The transition from adolescence to early adulthood is nevertheless commonly accompanied by a decline in physical activity as established routines, school-based opportunities and family structures are disrupted (2). For many young adults, entry into university introduces greater personal autonomy but also irregular timetables, academic pressure and substantial periods of sedentary study. A systematic review and meta-analysis estimated that university students spend more than 7 hours per day sedentary, highlighting the extent to which insufficient movement may become embedded within everyday university life (3). Because behavioural patterns established during this period may extend into later adulthood, universities represent an important setting for early, population-level physical activity promotion.

University-based health promotion has the potential to reach large and diverse groups of young adults through institutional policies, curricula, facilities and digital infrastructure. Unlike individually delivered counselling programmes, strategies embedded within routine university systems can be implemented across entire student populations with comparatively limited requirements for continuous staff contact. Institutional activity targets are one such strategy. By establishing a shared expectation, such targets may communicate the importance of regular participation and provide a common basis for monitoring progress. However, specifying how many activities students should complete does not necessarily enable them to decide when, where or how to act. Reviews and empirical studies among university students indicate that competing academic commitments, time constraints, low motivation, limited self-regulation and difficulties maintaining routines remain common barriers to sustained physical activity (4–6).

This distinction reflects a broader gap between behavioural intention and behavioural enactment. Students may recognise the value of physical activity and intend to participate, yet fail to translate that intention into action when suitable opportunities are not identified in advance or when planned activity is displaced by changing daily demands (7). A fixed institutional target may therefore establish an outcome expectation without providing sufficient support for the moment-to-moment organisation of behaviour. Approaches that assist students in identifying feasible opportunities, remembering intended actions and reviewing accumulated progress may be particularly relevant in university environments, where schedules vary across days and weeks and where the practical conditions for physical activity are rarely stable throughout an academic semester.

Digital health technologies offer a potentially scalable means of extending such behavioural support beyond face-to-face contact. Smartphone applications, activity-monitoring platforms, wearable devices and automated messaging systems can assist with goal setting, action planning, self-monitoring, prompts and feedback. Nevertheless, the presence of technology alone does not ensure behavioural change. Systematic reviews of digital interventions among college students and healthy young adults have reported small, inconsistent or outcome-specific effects (8–10). Some interventions have increased step counts or selected measures of activity, whereas effects on total physical activity, moderate-to-vigorous physical activity, sedentary behaviour and health-related outcomes have been less consistent. In a trial embedded in university physical activity courses, adding a wearable tracker did not increase physical activity (11). By contrast, a separate trial found higher step counts when cue-contingent financial incentives were combined with behavioural anchoring (12).

These mixed findings suggest that digital interventions should be evaluated according to the behavioural functions they deliver rather than treated as a single, homogeneous category. The effect of a digital strategy may depend on whether support is personally relevant, delivered at an actionable time and sufficiently integrated into the user’s daily context. Engagement is also important: an intervention cannot influence behaviour if its messages are poorly timed, repetitive, ignored or disconnected from the circumstances in which action is possible. Evidence from digital health research suggests that tailoring, reminders, feedback and other engagement strategies can improve interaction with digital interventions, although their effectiveness varies across populations and delivery conditions (13).

Intelligent assistants may address some of these limitations by coordinating several forms of behavioural support. In the present setting, the assistant was linked to the university timetable system and identified periods in which students had no scheduled classes. Rather than issuing reminders at a fixed time, it progressively incorporated information about each student’s recorded activity habits and preferred participation periods, and dynamically adjusted the timing of subsequent recommendations. Feedback was displayed immediately after a valid activity had been recorded and summarised accumulated participation, running sessions and running distance. These functions correspond broadly to action planning, prompts or cues, self-monitoring and feedback on behaviour within established behaviour change taxonomies (14).

The adaptive timing of support also shares features with just-in-time adaptive approaches, in which intervention content or timing is modified in response to information collected about an individual’s changing context or behaviour (15, 16). The aim is not simply to increase the volume of messages delivered, but to provide support when an individual is both available and potentially receptive to acting. In a university context, combining timetable-derived availability with progressively learned activity patterns may reduce the organisational burden of identifying feasible exercise periods. Immediate feedback may additionally make participation visible, reinforce awareness of progress and support subsequent planning. However, these mechanisms should not be assumed to produce uniformly beneficial outcomes. Repeated prompts may lose salience, students may disregard recommendations, and assistance with scheduling may influence the type or timing of activity without increasing its overall frequency.

The possibility that digital support may reorganise rather than simply increase activity has received comparatively limited attention. Physical activity is multidimensional. Participation frequency reflects how often activity occurs, but does not indicate what students do during those occasions. Activity composition reflects how total participation is distributed across available forms of movement, while session-level volume describes how much activity is accumulated after a particular activity has been initiated. These dimensions need not change in parallel. For example, students may complete a similar number of total sessions while allocating a greater proportion of them to running, or they may run slightly farther during each running session without increasing the number of occasions on which they are active.

Distinguishing these outcomes is important for both interpretation and programme evaluation. A change in activity composition may reveal that an intervention has influenced behavioural selection even when it has not increased total participation. At the same time, a greater proportion of running sessions should not automatically be interpreted as a superior outcome. It may reflect substitution away from other activities, differences in the types of activity recognised by the monitoring system or the relative ease with which particular activities satisfy institutional validity criteria. Similarly, a greater mean distance per running session does not establish that students exercised at a higher intensity or accumulated more health-enhancing activity overall. Evaluating multiple dimensions of recorded participation therefore provides a more informative account of how a digital support strategy operates, while requiring appropriately cautious interpretation.

Objective monitoring is particularly valuable in this context because retrospective self-reports are vulnerable to recall error and social desirability bias (17). Continuous institutional monitoring can capture participation repeatedly over an academic semester and link recorded activities to individual, de-identified accounts under routine conditions. It therefore offers an opportunity to evaluate how a strategy functions when delivered at scale rather than under tightly controlled experimental conditions. Previous work has shown that digital monitoring and immediate feedback can be integrated into university physical activity systems and used to examine objectively recorded participation and fitness outcomes (18).

Routine monitoring data nevertheless have important limitations. A monitoring system records only the activities and dimensions that it is designed to recognise. It may not capture unmonitored exercise, pace, physiological intensity, perceived exertion, social context or the reasons why students selected one activity rather than another. Recorded sessions therefore represent monitored participation rather than total physical activity. Real-world implementation data also typically provide less control over baseline equivalence, intervention exposure and contextual variation than randomised efficacy trials. Their value lies instead in assessing reach, identifying the behavioural dimensions most responsive under routine conditions and generating evidence to inform more rigorous pragmatic evaluations.

Evidence remains limited for university-wide approaches that integrate timetable-informed scheduling, dynamically adjusted reminders and immediate behavioural feedback while holding both the institutional activity target and objective monitoring environment constant across comparison groups. This comparative setting is important because it enables the additional contribution associated with intelligent support to be examined under a common institutional activity requirement and monitoring environment. It also reflects a realistic implementation question: whether existing institutional digital infrastructure can provide additional behavioural support to large numbers of students without requiring continuous one-to-one counselling.

The public health relevance of such an approach depends on more than statistical differences between groups. Population-level value is also shaped by reach, adoption, implementation fidelity, cost, sustainability and the equitable distribution of benefits (19–21). A strategy that produces only small individual differences may still warrant investigation when it can be delivered consistently across thousands of students at relatively low marginal cost. Conversely, large-scale availability does not guarantee meaningful engagement or equitable benefit. Students with different schedules, activity histories, socioeconomic circumstances or digital access may respond differently to the same support. Evaluation should therefore distinguish observed behavioural associations from broader claims about feasibility, effectiveness or scalability that require direct implementation evidence.

Against this background, the present study compared objectively recorded extracurricular physical activity patterns between students receiving intelligent-assistant support and those following usual self-scheduling during one academic semester. Both groups were assigned the same target of 45 extracurricular physical activity sessions and were monitored using identical procedures. The intelligent-assistant-supported group additionally received timetable-informed adaptive scheduling, dynamically timed reminders based on evolving activity habits and immediate post-activity feedback.

The main physical activity measures were total activity sessions, the proportion of sessions involving running and mean distance per running session. These outcomes were selected to distinguish overall participation frequency from activity composition and session-level running volume. Additional measures were end-of-semester body mass index, vital capacity and sex-specific endurance-run performance. By comparing the two support approaches within a shared institutional target and monitoring environment, this study sought to determine whether intelligent-assistant support was associated with differences in how students organised their extracurricular physical activity and whether any such differences were accompanied by more favourable anthropometric or fitness outcomes. Given the non-randomised design and the absence of baseline physical activity, body mass index and fitness measures, all between-group findings were interpreted as associations rather than causal effects.

## 2 Methods

### 2.1 Study design and setting

A semester-long comparative evaluation was conducted at a university in China. The study evaluated two approaches to supporting extracurricular physical activity under routine institutional conditions. Reporting was guided by the Transparent Reporting of Evaluations with Nonrandomized Designs statement, and the description of the support approaches was informed by the Template for Intervention Description and Replication checklist (22, 23).

The two support approaches operated within existing physical education course sections as part of routine institutional practice. The support approaches were organised within course sections rather than separately for individual students. Instructors generally taught multiple course sections, and the same instructors contributed course sections to both support approaches. Instructors were therefore not uniquely associated with one study condition, which reduced the likelihood that between-group differences were attributable solely to instructor characteristics or teaching practices.

Extracurricular physical activity was completed individually outside scheduled physical education classes. The study did not include class-based exercise sessions, unified extracurricular activity schedules, collective exercise supervision or additional face-to-face counselling. Once a support approach had been implemented within a course section, scheduling, reminders, participation and feedback operated at the individual student level.

### 2.2 Participants and analytic sample

Extracurricular physical activity was monitored from 1 September 2025 to 28 January 2026. The initial dataset contained records from 7,389 undergraduate students enrolled in the relevant physical education course sections during the monitoring period. The analytic sample was restricted to students for whom basic participant information, end-of-semester fitness assessment data and complete semester-long physical activity monitoring records were available.

All students were expected to complete 45 valid extracurricular physical activity sessions during the semester as part of the institutional physical activity requirement. Students in both groups were informed that their activity would be monitored. The monitoring period, activity-recognition procedures, validity criteria and institutional activity target were identical across the two groups. The principal difference between the groups was therefore the additional scheduling, reminder and feedback support available to the intelligent-assistant-supported group.

### 2.3 Physical activity support approaches

#### 2.3.1 Intelligent-assistant support

The intelligent-assistant platform functioned as the scheduling, notification and feedback interface. It did not itself identify or measure physical activity; activity recognition was undertaken by the separate motion-behaviour monitoring terminals described below. Once a student’s activity had been recognised and validated by the monitoring system, the resulting record was transmitted to the platform for feedback and subsequent scheduling support.

The platform was connected to the university academic affairs system and accessed each participating student’s class timetable within the operational university information environment. Periods without scheduled teaching were identified as potentially available windows for extracurricular physical activity. Timetable information was used for operational scheduling within the university system and was not included in the de-identified research dataset.

The platform did not issue reminders at a single fixed or uniformly prespecified time. As a student accumulated valid physical activity records, the system progressively updated an individual temporal participation profile reflecting when that student tended to undertake extracurricular activity. An adaptive scheduling algorithm combined timetable-defined availability with this evolving profile to identify recommended activity periods and dynamically adjust the timing of subsequent reminders. The timing of support could therefore change across the semester in response to the student’s recorded participation pattern.

Immediately after a valid activity session had been confirmed by the monitoring system, feedback was made available through the mobile application. The feedback displayed the student’s accumulated number of valid activity sessions, number of running sessions and accumulated running distance. Students could also use a computer-based dashboard to view more detailed summaries of their recorded activity. Feedback was generated from validated monitoring records rather than from self-reported activity.

The intelligent-assistant-supported approach therefore combined three principal behavioural support functions: timetable-informed action planning, dynamically timed prompts or cues and immediate feedback on recorded behaviour. These components were delivered as an integrated support strategy, and the independent contribution of each component was not evaluated. The platform did not prescribe an individualised activity type, exercise intensity, running pace, session duration or training load.

Personalisation was confined to the timing of recommended participation and was based on the interaction between timetable availability and the student’s evolving temporal activity pattern. The approach was consequently defined as intelligent-assistant support for physical activity participation rather than as individualised exercise prescription. It was designed to reduce the organisational demands associated with identifying feasible activity opportunities, rather than to replace professional exercise programming or instruction.

#### 2.3.2 Usual self-scheduling

Students in the usual self-scheduled group were subject to the same institutional target of 45 valid extracurricular physical activity sessions and the same objective monitoring procedures. They independently determined when to undertake extracurricular physical activity. They did not receive automated timetable matching, algorithmically adjusted reminder timing or immediate post-activity feedback through the intelligent-assistant interface.

No additional restrictions were imposed on the types of valid activity that students in either group could select. The comparison was therefore between intelligent assistance for organising participation and usual self-scheduling under a shared institutional target and monitoring environment, rather than between different exercise prescriptions (Figure 1).

**Figure 1:**
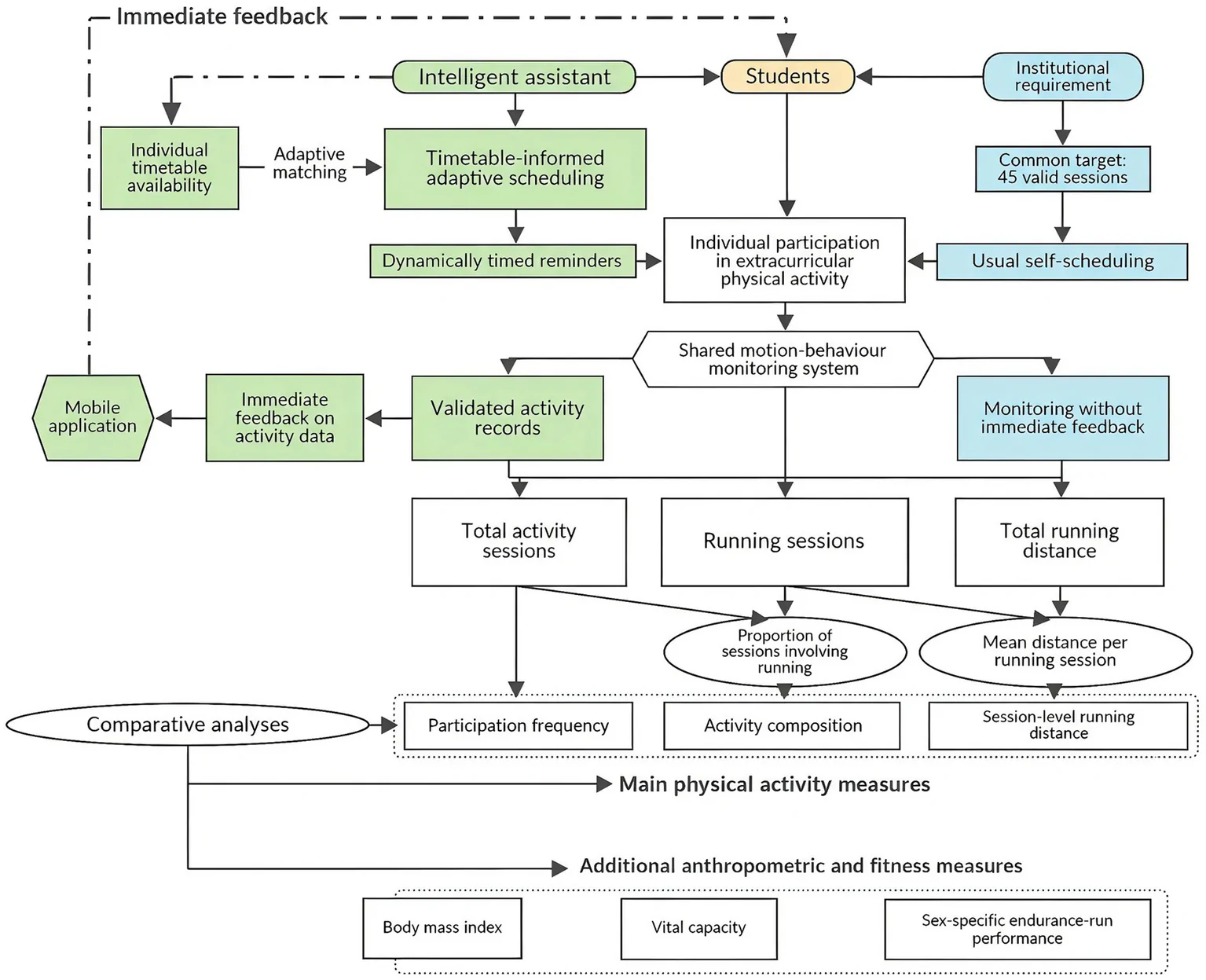
Overview of intelligent-assistant support, usual self-scheduling and the shared monitoring system.

### 2.4 Physical activity monitoring

Extracurricular physical activity was captured using a non-contact, wearable-free motion-behaviour monitoring system deployed through institutional monitoring terminals. The monitoring terminals used face-based identity verification to associate an observed activity with the appropriate registered student account. Recognised activity data were then transmitted to the system backend for processing and validation. The intelligent-assistant platform displayed the resulting activity records but did not itself perform activity recognition.

The same monitoring terminals, identity-verification procedures, activity-recognition processes and backend validation rules were applied to both groups throughout the monitoring period. Face-based verification was used to reduce the possibility that one student could complete or register an activity on behalf of another. Identity verification was conducted within the university’s operational information system and did not provide the research team with facial images or biometric templates.

A valid extracurricular physical activity session was defined using activity-specific criteria established before the monitoring period and communicated to all students. For example, a running session was required to include a recorded distance of at least 2 km, whereas a ball-sport session was required to include at least 60 min of recorded participation. These thresholds formed part of the routine institutional rules for recognising a completed extracurricular physical activity session.

Predefined backend validation procedures were used to identify duplicate records, records that did not meet the activity-specific distance or duration threshold and other implausible or invalid records. Records that failed the applicable validation criteria were not counted as valid sessions. The same criteria were applied consistently to both support groups.

Each validated activity session was linked to a unique registered account within the operational system. Before data were released for research, direct identifiers were removed and the account was represented by a de-identified identifier. The analytic dataset therefore permitted repeated activity records to be linked to the same student account without revealing the student’s identity to the research team.

### 2.5 Outcome measures

#### 2.5.1 Physical activity outcomes

Available activity records included the total number of valid extracurricular physical activity sessions, the number of running sessions, accumulated running distance and the number of sessions involving other recognised forms of activity. The total number of activity sessions represented the accumulated frequency of objectively recorded valid extracurricular physical activity during the semester.

The proportion of sessions involving running was calculated by dividing the number of running sessions by the total number of valid activity sessions and multiplying the result by 100. This derived measure represented the proportion of monitored participation allocated to running. It was interpreted as an indicator of recorded activity composition rather than as a measure of total physical activity volume or exercise preference.

Mean distance per running session was calculated by dividing accumulated running distance by the number of valid running sessions. It represented the distance accumulated during an average recorded running session. Every student included in the analytic sample had at least one valid running session; consequently, this outcome could be calculated for the full analytic sample without assigning an artificial value to students with no recorded running.

These indicators described activity recorded within the institutional monitoring environment. They were not interpreted as direct measures of exercise motivation, subjective preference, physiological intensity, perceived exertion or activity quality. The system did not record running pace, physiological workload or physical activity undertaken outside the monitored environment. Baseline physical activity data were unavailable, so the semester-long records could not establish changes from pre-existing activity levels.

#### 2.5.2 Anthropometric and fitness outcomes

End-of-semester assessments were conducted by trained staff using institutional fitness-testing equipment (HK6800 series; Hengkang Jiaye). The available measures were height, weight, vital capacity and endurance-run performance. Only end-of-semester measurements were available in the research dataset; baseline body mass index and fitness measurements were unavailable, precluding assessment of within-person change.

Body mass index was calculated by dividing weight in kilograms by height in metres squared and was analysed as a continuous variable. Vital capacity was recorded in millilitres. These outcomes were treated as end-of-semester anthropometric and fitness indicators rather than as measures of change during the semester.

Endurance performance was assessed using the university’s sex-specific testing protocol. Female students completed an 800-m run and men completed a 1,000-m run. Values were initially stored using minutes-and-seconds notation in the form M. SS. Before analysis, each record was converted to total seconds by multiplying the integer-minute component by 60 and adding the two-digit seconds component. Lower values indicated faster endurance-run performance.

Because female and male students completed different running distances, endurance-run outcomes were analysed separately by sex. The raw 800-m and 1,000-m times were not pooled into a single outcome, because such pooling would combine performances obtained from different test protocols.

### 2.6 Data management and privacy protection

Timetable matching, activity recognition and operational identity verification were conducted within the university information environment. The research team received only a de-identified analytic extract. The extract contained an account identifier that allowed repeated records to be linked within the dataset but did not contain names, student numbers, facial images, biometric templates, individual timetable details, course-section identifiers or direct links to student identities.

The removal of course-section identifiers protected against the re-identification of individual students through combinations of timetable, class and activity information. It also meant that the analytic dataset could not be used to model potential statistical dependence among students within the same course section. The participant account was therefore the unit of analysis.

### 2.7 Statistical analysis

Participant characteristics were summarised using means and standard deviations for continuous variables and frequencies and percentages for categorical variables. Because the distributions of the physical activity outcomes were skewed, these outcomes were summarised using medians and interquartile ranges. End-of-semester anthropometric and fitness outcomes were summarised using means and standard deviations.

Unadjusted between-group differences in total activity sessions, the proportion of sessions involving running and mean distance per running session were examined using two-sided Mann–Whitney U tests. Rank-biserial correlations were calculated to describe the magnitude and direction of the corresponding distributional differences. Non-parametric 95% confidence intervals for the rank-biserial correlations were obtained from 1,000 bootstrap resamples.

No separate comparison of any versus no running was conducted because every participant in both groups recorded at least one valid running session; this indicator therefore showed no between-group variation.

Covariate-adjusted absolute mean differences between the intelligent-assistant-supported and usual self-scheduled groups were estimated using ordinary least-squares regression with HC3 heteroscedasticity-consistent standard errors. Study group was entered as an indicator variable, with the usual self-scheduled group serving as the reference category. Positive estimates indicated higher values in the intelligent-assistant-supported group, whereas negative estimates indicated lower values.

Models for total activity sessions, the proportion of sessions involving running, mean distance per running session and body mass index were adjusted for sex and academic year. The vital-capacity model was adjusted for sex, academic year, height and weight. Endurance-run models were stratified by sex and adjusted for academic year and body mass index. Because the anthropometric and fitness covariates were measured at the end of the semester, the resulting coefficients were interpreted as covariate-adjusted descriptive associations and not as causal intervention effects.

### 2.8 Ethical approval and informed consent

This study was approved in June 2022 by the Research Ethics Committee of CQUPT. Approval number is 2022111002696. Electronic informed consent was obtained from all participants before the monitoring period began. Participants were informed that their extracurricular physical activity would be monitored throughout the semester and that recorded activity and fitness data would be used for research purposes. Operational timetable matching and identity verification remained within the university information systems, and the research team analysed only the de-identified dataset described above.

## 3 Results

### 3.1 Participant flow and characteristics

Of the 7,389 undergraduate records screened, 357 had missing basic participant information, 678 lacked end-of-semester fitness assessment data and 124 had incomplete semester-long physical activity monitoring records. Because these exclusion categories were not mutually exclusive, the category-specific counts exceeded the number of unique records excluded. After 859 unique records meeting at least one exclusion criterion had been removed, the final analytic sample comprised 6,530 students, including 3,258 in the intelligent-assistant-supported group and 3,272 in the usual self-scheduled group (Table 1).

**Table 1:** Participant characteristics and end-of-semester anthropometric measures.

Characteristic	Intelligent-assistant group	Usual self-scheduling group	Total
Participants, *n*	3,258	3,272	6,530
Female students, *n* (%)	819 (25.1)	844 (25.8)	1,663 (25.5)
Male students, *n* (%)	2,439 (74.9)	2,428 (74.2)	4,867 (74.5)
Year 1, *n* (%)	1,183 (36.3)	1,251 (38.2)	2,434 (37.3)
Year 2, *n* (%)	1,069 (32.8)	1,121 (34.3)	2,190 (33.5)
Year 3, *n* (%)	932 (28.6)	841 (25.7)	1773 (27.2)
Year 4, *n* (%)	74 (2.3)	59 (1.8)	133 (2.0)
Height, m, mean (SD)	1.698 (0.078)	1.700 (0.079)	1.699 (0.078)
Weight, kg, mean (SD)	63.4 (11.9)	62.4 (11.4)	62.9 (11.7)
BMI, kg/m^2^, mean (SD)	21.90 (3.33)	21.52 (3.15)	21.71 (3.25)

Female students accounted for 25.5% of the overall sample and men for 74.5%. The proportion of female students was 25.1% in the intelligent-assistant-supported group and 25.8% in the usual self-scheduled group. Mean height was 1.698 m and 1.700 m, respectively. The intelligent-assistant-supported group included a larger proportion of third-year students than the usual self-scheduled group (28.6% versus 25.7%) and had higher mean end-of-semester weight and body mass index.

### 3.2 Extracurricular physical activity patterns

All students included in the analytic sample recorded at least one valid running session during the semester. The intelligent-assistant-supported group completed a median of 38 valid extracurricular physical activity sessions (interquartile range, 36–40), compared with 39 sessions (interquartile range, 36–41) in the usual self-scheduled group (Table 2). The rank-biserial correlation was −0.115.

**Table 2:** Extracurricular physical activity outcomes by support approach.

Outcome	Intelligent-assistant group	Usual self-scheduling group	Rank-biserial correlation (95% CI)	Adjusted difference (95% CI)	p
Total activity sessions	38 (36–40)	39 (36–41)	−0.115 (−0.141 to −0.089)	−0.58 (−0.72 to −0.43)	<0.001
Sessions involving running, % of total	64.71 (61.90–67.50)	63.41 (60.98–65.79)	0.179 (0.151 to 0.206)	+2.13 pp. (1.83 to 2.44)	<0.001
Mean distance per running session, km	2.128 (1.968–2.246)	2.042 (1.946–2.197)	0.158 (0.130 to 0.185)	+0.061 (0.051 to 0.071)	<0.001

In the covariate-adjusted model, students receiving intelligent-assistant support recorded 0.58 fewer activity sessions than those following usual self-scheduling (*p* < 0.001). The median number of sessions in both groups was below the common institutional target of 45 valid extracurricular physical activity sessions.

The median proportion of sessions involving running was 64.71% (interquartile range, 61.90–67.50%) in the intelligent-assistant-supported group and 63.41% (interquartile range, 60.98–65.79%) in the usual self-scheduled group. The rank-biserial correlation was 0.179 (95% confidence interval, 0.151 to 0.206). After adjustment for sex and academic year, the proportion of sessions involving running was 2.13 percentage points higher in the intelligent-assistant-supported group (*p* < 0.001).

Median distance per running session was 2.128 km (interquartile range, 1.968–2.246 km) in the intelligent-assistant-supported group and 2.042 km (interquartile range, 1.946–2.197 km) in the usual self-scheduled group. The rank-biserial correlation was 0.158 (95% confidence interval, 0.130 to 0.185). In the adjusted model, mean distance per running session was 0.061 km greater in the intelligent-assistant-supported group (*p* < 0.001), corresponding to approximately 61 m per running session.

Overall, intelligent-assistant support was associated with slightly fewer recorded activity sessions but a greater proportion of sessions involving running and a marginally greater distance per running session.

### 3.3 End-of-semester anthropometric and fitness outcomes

Mean end-of-semester body mass index was 21.90 kg/m^2^ in the intelligent-assistant-supported group and 21.52 kg/m^2^ in the usual self-scheduled group (Table 3). After adjustment for sex and academic year, body mass index was 0.36 kg/m^2^ higher in the intelligent-assistant-supported group (*p* < 0.001).

**Table 3:** End-of-semester anthropometric and fitness outcomes by support approach.

Outcome	Intelligent-assistant group, mean (SD)	Usual self-scheduling group, mean (SD)	Adjusted difference (95% CI)	*p*
BMI, kg/m^2^	21.90 (3.33)	21.52 (3.15)	+0.36 (0.21 to 0.52)	<0.001
Vital capacity, mL	3115.9 (712.6)	3063.5 (687.7)	+41.4 (17.5 to 65.3)	<0.001
800-m endurance-run time, female students, s	248.2 (22.9)	247.8 (20.7)	−0.18 (−2.29 to 1.92)	0.864
1,000-m endurance-run time, male students, s	257.0 (27.7)	258.5 (24.4)	−2.68 (−4.04 to −1.32)	<0.001

Note. Differences are calculated as intelligent-assistant-supported minus usual self-scheduled. BMI models included sex and academic year. Vital-capacity models additionally included height and weight. Endurance-run models were stratified by sex and included academic year and BMI. Lower endurance-run time indicates faster performance.

Mean vital capacity was 3,115.9 mL in the intelligent-assistant-supported group and 3,063.5 mL in the usual self-scheduled group. After adjustment for sex, academic year, height and weight, vital capacity was 41.4 mL higher in the intelligent-assistant-supported group (*p* < 0.001).

Among female students, mean 800-m endurance-run time was 248.2 s in the intelligent-assistant-supported group and 247.8 s in the usual self-scheduled group. After adjustment for academic year and body mass index, the between-group difference was −0.18 s (*p* = 0.864).

Among male students, mean 1,000-m endurance-run time was 257.0 s in the intelligent-assistant-supported group and 258.5 s in the usual self-scheduled group. After adjustment for academic year and body mass index, endurance-run time was 2.68 s lower in the intelligent-assistant-supported group (*p* < 0.001).

The anthropometric and fitness findings were not consistent across outcomes. Intelligent-assistant support was associated with higher vital capacity and faster endurance-run performance among male students, but no difference in endurance-run performance among female students and a higher end-of-semester body mass index.

## 4 Discussion

### 4.1 Principal findings and contribution

This semester-long, non-randomised study evaluated whether intelligent-assistant support provided additional value beyond an existing university physical activity programme in which all students received the same target of 45 extracurricular activity sessions and identical objective monitoring. Intelligent-assistant support was not associated with a higher frequency of recorded participation. Instead, students receiving the support completed slightly fewer sessions, although they allocated a greater proportion of their recorded activities to running and completed a marginally greater distance per running session. Differences in anthropometric and fitness outcomes were small and inconsistent. Taken together, these findings indicate that the principal association of intelligent-assistant support was with the organisation and composition of recorded activity rather than with greater overall participation or a consistent fitness advantage.

This pattern adds a distinct perspective to the digital physical activity literature. Most previous evaluations have treated physical activity change primarily as an increase or decrease in aggregate outcomes such as step count, minutes of moderate-to-vigorous physical activity, total activity time or sedentary time. The present study suggests that this emphasis may overlook another possible response to digital support: participation can be redistributed among activity types even when the overall number of participation occasions remains largely unchanged. The study therefore extends the evaluative focus from whether digital support increases physical activity to how it may shape the way in which activity is selected and organised.

### 4.2 Intelligent-assistant support and overall participation frequency

The absence of a higher total session count is broadly consistent with evidence that digital physical activity interventions do not exert uniform effects across behavioural outcomes. A recent meta-analysis restricted to college students found that digital health interventions increased objectively measured step counts but did not produce consistent changes in light physical activity, moderate-to-vigorous physical activity or sedentary time (8). A separate meta-analysis of e-health interventions among college students similarly reported substantial heterogeneity across intervention formats and outcomes (24). Reviews covering broader adult populations have found small-to-moderate average effects of smartphone applications and activity trackers, but these effects varied according to personalisation, messaging features, intervention duration and the physical activity measure selected (10, 25).

At the same time, the present findings differ from studies and reviews reporting increases in physical activity following applications, text messaging, social media interventions or wearable tracking (25, 26). This apparent inconsistency is likely to reflect differences in the comparison condition. Many previous trials compared a multicomponent digital intervention with no intervention, usual lifestyle or the provision of basic health information. In the present study, both groups were already exposed to an institutional activity requirement, objective monitoring and knowledge that their participation was being recorded. Intelligent-assistant support therefore represented an incremental addition to an active institutional programme, rather than the entire contrast between behavioural support and no support. The common target and monitoring environment may have reduced the amount of additional behavioural change that scheduling and feedback could generate.

This interpretation is supported by a cluster-randomised trial conducted within university physical activity courses, in which adding a wearable activity tracker to an existing course did not increase overall physical activity (11). By contrast, interventions that combine digital monitoring with additional elements such as explicit behavioural goals, incentives, coaching or social support have sometimes produced more favourable outcomes (12, 25, 26). The comparison suggests that digital monitoring and reminders may be more effective when they form part of a broader behaviour change programme than when they are added to an environment that already defines participation expectations.

Differences in outcome definition are also important. Meta-analyses reporting favourable effects frequently use step count or accumulated activity minutes, whereas the present study measured the number of institutionally valid activity sessions. A student could increase the amount of movement within daily life without completing an additional valid session, while an additional session could meet the institutional threshold without indicating a large change in weekly energy expenditure. The absence of a higher session count should therefore not be regarded as direct evidence that intelligent-assistant support had no effect on total physical activity. The observed comparison shows no higher frequency of activities recognised as valid within the university monitoring system among students receiving the support.

The finding that median participation remained below the target in both groups further indicates that scheduling support addressed only one component of a multidetermined behaviour. Linking recommendations to timetable availability may reduce the practical difficulty of identifying when to exercise, but it does not directly address motivation, fatigue, academic workload, access to facilities, weather, social support or preferences for different forms of activity. Reviews of university physical activity promotion consistently identify multiple interacting barriers rather than a single time-management problem (4). The current findings therefore refine, rather than reject, the value of adaptive scheduling: identifying an available time may be necessary for participation, but it is not sufficient to ensure that participation occurs.

The slightly lower session count in the intelligent-assistant-supported group also contrasts with the expectation that more appropriately timed reminders should increase participation. Several explanations are possible. Recommendations may have displaced students’ own plans without increasing the total number of opportunities used; repeated prompts may have lost salience over the semester; or students may have engaged with the platform selectively. However, because reminder delivery, opening and adherence were not retained in the analytic dataset, these explanations cannot be tested. Mobile health research has repeatedly shown that intervention availability does not guarantee sustained engagement and that attrition or declining use can substantially weaken the delivered intervention dose (13).

Accordingly, the result should not be interpreted as evidence that intelligent-assistant support reduced participation. The adjusted difference was less than one session over an entire semester, and unmeasured baseline differences remain possible in a non-randomised design. The more defensible conclusion is that the additional digital functions were not associated with higher recorded participation frequency under the common institutional programme.

### 4.3 Intelligent-assistant support and the reorganisation of activity composition

The higher running share and marginally greater distance per running session provide the most distinctive finding of the study. Existing digital physical activity reviews have generally examined whether interventions increase steps, activity minutes or intensity categories (8–10, 24, 25). They provide comparatively limited evidence about whether digital support changes the distribution of participation across different activities. By separating total session frequency from the proportion of sessions involving running, the present study shows that digital support can be associated with behavioural reallocation even when the aggregate number of participation occasions does not increase.

This finding is consistent with evidence that digital tracking tends to influence behaviours that are deliberately initiated, readily quantified and immediately visible to the user. Li and colleagues found that wearable activity trackers improved daily steps and weekly moderate-to-vigorous physical activity, but not light physical activity or sedentary behaviour, and interpreted this pattern as a stronger effect on consciously monitored exercise than on habitual behaviour (9). Running distance is similarly discrete, measurable and easily displayed as feedback. The present findings therefore support the proposition that digital systems may exert their strongest influence on behaviours that can be clearly represented through counts, distances and progress indicators.

The relationship between the platform and running was nevertheless indirect. The intelligent assistant did not prescribe running, and students remained free to choose among valid activity types. One possible explanation is that running was easier to fit into individually available timetable windows than activities requiring partners, teams or specific facility bookings. This interpretation would be compatible with the function of the assistant: it was designed to reduce uncertainty about when an individual could act, and independent running may have been especially responsive to that form of support. By comparison, participation in ball sports may depend more heavily on the simultaneous availability of other students and facilities, constraints that an individual timetable-matching algorithm cannot resolve.

A second explanation concerns the visibility created by monitoring and feedback. The platform displayed the number of running sessions and accumulated running distance immediately after participation. When one activity produces clearer and more detailed feedback than others, the monitoring system may increase the salience of that activity and make progress easier to recognise. In this sense, digital technology may not function as a neutral channel that merely records pre-existing preferences. Its measurement categories and feedback displays may shape which behaviours attract attention and are subsequently repeated. The higher running share may therefore reflect not only support for participation, but also the behavioural influence of what the system makes visible.

This interpretation also introduces an important qualification to claims of personalisation. The assistant personalised the timing of recommendations, but it did not personalise activity type or exercise dose. An intervention can therefore be personalised in one dimension while simultaneously encouraging behavioural convergence in another. Students received different recommended times, yet the activity pattern associated with the support was a slightly greater concentration on the same measurable activity. This distinction is relevant to digital health promotion because personalisation of delivery should not automatically be equated with diversification or individualisation of behavioural outcomes.

The adaptive reminder process shares features with just-in-time adaptive interventions, which seek to provide support in response to changing information about an individual’s context and receptivity. Existing reviews suggest that context-sensitive prompts may be acceptable and can increase the perceived relevance of messages, but evidence of behavioural effectiveness remains mixed. Technical reliability, the accuracy of decision rules and the actual timeliness of messages are recurring challenges (15). The present results are compatible with the possibility that adaptive timing influenced activity selection, but the study cannot establish that it was more effective than fixed reminders because no fixed-reminder comparison was included and no proximal response to individual messages was measured.

The absolute magnitude of the running-related differences must also remain central to interpretation. A 2.13-percentage-point difference in running share and approximately 61 m per running session are small between-group differences. Neither measure captures running pace, physiological intensity or total weekly running volume, and a higher proportion is mathematically compatible with fewer non-running sessions. Consequently, the findings identify a difference in recorded activity organisation, but they do not show that the supported group accumulated a meaningfully greater dose of health-enhancing activity. The contribution is conceptual and behavioural rather than evidence of physiological superiority.

### 4.4 Anthropometric and fitness outcomes in relation to previous evidence

The mixed fitness findings are broadly consistent with reviews showing that digital physical activity interventions produce more reliable changes in proximal behavioural outcomes than in downstream physiological outcomes. The umbrella review by Ferguson and colleagues found favourable average effects of wearable trackers on physical activity, but effects on physiological outcomes were generally smaller and varied across measures and populations (10). College-student reviews have likewise reported clearer evidence for selected activity measures than for broader health outcomes (8, 24). The present study follows this pattern: the most coherent differences occurred in objectively recorded behaviour, whereas vital capacity, endurance performance and body mass index did not form a consistent set of favourable outcomes.

This result differs from some digital intervention studies that have reported improvements in weight-related or fitness outcomes. Such differences may reflect the intensity and content of the intervention. Programmes that affect body weight or cardiorespiratory fitness commonly include explicit exercise prescriptions, progressive training targets, dietary components, coaching or sustained increases in total activity volume. The intelligent assistant evaluated here was deliberately narrower: it recommended when activity might be undertaken but did not prescribe the activity type, intensity, pace, duration or progression. Scheduling support cannot be expected to produce the same physiological response as a structured exercise-training or weight-management programme if it does not generate a sufficient difference in training dose.

The higher adjusted vital capacity and faster 1,000-m performance among male students should be interpreted against the size and consistency of the estimates. The vital-capacity difference was small relative to the within-group variability, and the difference in male students’ endurance time was less than 3 s. The absence of a corresponding difference in female students does not demonstrate a male-specific effect: statistical significance in one sex and non-significance in another is not itself evidence that the associations differ by sex. Moreover, female and male students completed different endurance tests. Without baseline data, a standardised common outcome and a formal interaction analysis, the study cannot determine whether sex modified the association between intelligent-assistant support and endurance performance.

The body mass index finding also differs from the favourable direction often anticipated in physical activity promotion. However, only end-of-semester measurements were available, and body mass index does not distinguish adipose tissue from lean mass. The higher value may represent a pre-existing group difference rather than a change during the semester. It should therefore not be described as either a harmful effect of the support or evidence of improved muscularity. Critical reviews have long cautioned that body mass index is a screening index rather than a direct measure of body composition or individual health status (27).

More fundamentally, baseline physical activity, body mass index and fitness data were unavailable, precluding assessment of within-person change and adjustment for pre-existing differences in these measures. Adjustment for measured characteristics cannot establish that the groups entered the semester with comparable vital capacity, endurance or body mass index. The anthropometric and fitness outcomes should therefore be regarded as secondary descriptive associations at the end of the semester. Their small and inconsistent differences do not establish a meaningful fitness benefit or a harmful effect of intelligent-assistant support.

### 4.5 Public health and practical implications

Statistical significance should be distinguished from practical or public health importance. In a sample of 6,530 students, small differences can yield small *p* values; moreover, unaccounted-for course-section clustering may overstate their precision. The adjusted difference of 0.58 fewer sessions represents less than one recorded activity occasion across the whole semester. A 2.13-percentage-point higher running share and 0.061 km (approximately 61 m) greater distance per running session indicate modest differences in activity composition and session-level distance, without establishing a greater total activity dose or intensity. Similarly, the 41.4-mL vital-capacity difference was small relative to mean values above 3,000 mL, and the 2.68-s difference in male students’ 1,000-m time was less than 3 s. The 0.36-kg/m^2^ higher body mass index does not establish either benefit or harm. These magnitudes therefore do not establish a meaningful health benefit, and their practical and public health importance remains uncertain.

The public health case for further evaluation therefore rests on potential integration into routine university infrastructure, while requiring evidence that any small behavioural differences translate into meaningful and sustained benefits. Intelligent-assistant support was delivered to more than 3,000 students through existing timetable and monitoring systems without continuous one-to-one counselling. Digital interventions can be attractive at the population level because the marginal cost of reaching an additional user may be lower than that of individually delivered support. Nevertheless, large reach does not compensate for uncertain effectiveness, and a small statistically significant association should not be treated as population benefit unless it is reproducible, sustained and achieved with acceptable cost and equity.

Implementation frameworks make this distinction explicit. RE-AIM separates reach from effectiveness, adoption, implementation and maintenance, while the PRACTIS guide emphasises contextual fit, delivery capacity, stakeholder engagement, barriers and sustainability when physical activity interventions are integrated into practice (19–21). The present study demonstrates reach and routine delivery, but it did not measure platform adoption, reminder exposure, fidelity, cost, maintenance or differential benefit across student groups. It should therefore be viewed as an outcome evaluation conducted under real-world conditions, rather than as a complete implementation or scale-up evaluation.

The findings also suggest that intelligent-assistant support should be positioned as one component of a multilevel university physical activity strategy. Adaptive scheduling may help students identify feasible opportunities, but the persistent shortfall from the institutional target indicates that participation is also shaped by motivation, social and environmental opportunities, facility provision and the design of the activity requirement itself. Combining digital support with a broader range of accessible activities, social support, targeted follow-up for students with low participation and appropriate institutional provision may be more promising than expecting a scheduling algorithm to operate as a stand-alone solution.

### 4.6 Strengths and limitations

This study has several strengths. It evaluated an adaptive digital support strategy in a large student population under routine university conditions, rather than in a small, tightly controlled trial. Both groups received identical activity targets, monitoring procedures and validity criteria, enabling the comparison to focus on the additional scheduling, reminder and feedback functions. Activity was objectively recorded over a complete semester, and the analysis distinguished participation frequency from activity composition and session-level running distance. This multidimensional outcome structure made it possible to identify a behavioural difference that would have been obscured by an analysis based only on total sessions.

The non-randomised design remains the principal limitation. Support conditions followed existing course-section arrangements, and baseline physical activity, body mass index and fitness measures were unavailable. The analyses could therefore neither adjust for pre-existing differences in these measures nor establish within-person change during the semester. Observed end-of-semester differences may reflect pre-existing group differences rather than changes associated with the support. Although the same instructors contributed sections to both conditions, other differences in student composition, timetable structure or course sections may remain. Removal of course-section identifiers also prevented adjustment for within-section correlation. Positive within-section correlation could lead to underestimated standard errors, overly narrow confidence intervals and overly small *p* values; the extent of this uncertainty could not be assessed. Covariate adjustment and a large sample cannot remove these limitations, and the reported associations should not be interpreted as causal effects.

Finally, actual exposure to and engagement with the intelligent-assistant functions could not be quantified in the present analysis. Adaptive scheduling, reminders and feedback were provided as an integrated package, but the analytic dataset did not include implementation logs on message generation or reminder delivery, message viewing, recommendation uptake, feedback use or the operational performance of the adaptive algorithm. These measures were therefore not analysed. The study could not assess whether associations varied with engagement or algorithm performance, or distinguish the contribution of individual support functions. Future prospective studies should retain de-identified course-section identifiers, collect baseline and follow-up physical activity, body mass index and fitness measures, and incorporate detailed implementation logs. Comparisons between adaptive and fixed reminder timing, potentially using micro-randomised designs, would be particularly useful for establishing whether dynamic timing provides benefits beyond simpler automated support (28).

## 5 Conclusion

This study evaluated intelligent-assistant support not as a stand-alone digital programme, but as an additional layer within an existing university physical activity system characterised by a common participation target and objective monitoring. Under these conditions, intelligent-assistant support was not associated with greater overall participation frequency. Its more discernible association was with the way recorded activity was organised, particularly the relative allocation of participation to running and the distance completed within running sessions. The anthropometric and fitness findings did not converge towards a consistent pattern of benefit. The evidence therefore does not support a simple conclusion that intelligent-assistant support increased physical activity or improved physical fitness.

The principal contribution of the study is to show that the behavioural influence of digital support may be expressed through reorganisation rather than expansion. Digital interventions are commonly evaluated according to whether they increase total steps, activity time or participation frequency. The present findings indicate that such aggregate outcomes may provide only a partial account of behavioural change. A digital system may leave the overall number of activity occasions largely unchanged while influencing which activities are selected, how participation is distributed and which forms of behaviour become most visible through monitoring and feedback. Evaluation of intelligent physical activity support should therefore distinguish participation frequency, activity composition, session-level volume and subsequent health outcomes, rather than treating them as interchangeable indicators of effectiveness.

This distinction also refines the practical role that intelligent assistants can reasonably play in university health promotion. Timetable-informed recommendations, adaptive reminders and immediate feedback may help reduce the organisational burden of identifying feasible opportunities and may provide greater structure to otherwise self-managed participation. However, they do not directly address the full range of motivational, social, environmental and resource-related barriers that determine sustained physical activity. Intelligent-assistant support should therefore be positioned as a complementary component of a broader institutional strategy, alongside accessible facilities, varied activity opportunities, social support and targeted assistance for students with persistently low participation. Its value lies in supporting the organisation of behaviour, not in replacing comprehensive physical activity promotion or professional exercise guidance.

The present findings should nevertheless be treated as evidence from routine implementation rather than as proof of causal effectiveness. A stronger evaluation will require prospective course-section-level designs, baseline and follow-up measurement, retention of de-identified clustering information, and direct assessment of recommendation delivery, reminder exposure, feedback use and engagement over time. Future studies should also examine whether changes in activity composition are accompanied by meaningful differences in total activity dose, intensity, fitness, sustainability and equity across student groups. Only when behavioural, implementation and public health outcomes are evaluated together will it be possible to determine whether intelligent-assistant support offers a meaningful advantage over simpler forms of digital or institutional support.

## Data Availability

The de-identified data supporting the conclusions of this article will be made available by the authors upon reasonable request, subject to institutional data-protection requirements and the conditions of the ethical approval.

## References

[1] Bull FC Al-Ansari SS Biddle S Borodulin K Buman MP Cardon G . World Health Organization 2020 guidelines on physical activity and sedentary behaviour. Br J Sports Med. (2020) 54:1451–62. doi: 10.1136/bjsports-2020-102955, 33239350 PMC7719906

[2] Corder K Winpenny E Love R Brown HE White M van Sluijs EMF. Change in physical activity from adolescence to early adulthood: a systematic review and meta-analysis of longitudinal cohort studies. Br J Sports Med. (2019) 53:496–503. doi: 10.1136/bjsports-2016-097330, 28739834 PMC6250429

[3] Castro O Bennie J Vergeer I Bosselut G Biddle SJH. How sedentary are university students? A systematic review and meta-analysis. Prev Sci. (2020) 21:332–43. doi: 10.1007/s11121-020-01093-8, 31975312

[4] Johannes C Roman NV Onagbiye SO Titus S Leach LL. Strategies and best practices that enhance the physical activity levels of undergraduate university students: a systematic review. Int J Environ Res Public Health. (2024) 21:173. doi: 10.3390/ijerph21020173, 38397664 PMC10888190

[5] Maselli M Ward PB Gobbi E Carraro A. Promoting physical activity among university students: a systematic review of controlled trials. Am J Health Promot. (2018) 32:1602–12. doi: 10.1177/0890117117753798, 29366334

[6] Gómez-López M Gallegos AG Extremera AB. Perceived barriers by university students in the practice of physical activities. J Sports Sci Med. (2010) 9:374. PMC3761719 24149629

[7] Sheeran P Webb TL. The intention–behavior gap. Soc Personal Psychol Compass. (2016) 10:503–18. doi: 10.1111/spc3.12265

[8] Bi S Yuan J Wang Y Zhang W Zhang L Zhang Y . Effectiveness of digital health interventions in promoting physical activity among college students: systematic review and meta-analysis. J Med Internet Res. (2024) 26:e51714. doi: 10.2196/51714, 39566049 PMC11618011

[9] Li C Chen X Bi X. Wearable activity trackers for promoting physical activity: a systematic meta-analytic review. Int J Med Inform. (2021) 152:104487. doi: 10.1016/j.ijmedinf.2021.104487, 34020170

[10] Ferguson T Olds T Curtis R Blake H Crozier AJ Dankiw K . Effectiveness of wearable activity trackers to increase physical activity and improve health: a systematic review of systematic reviews and meta-analyses. Lancet Digit Health. (2022) 4:e615–26. doi: 10.1016/S2589-7500(22)00111-X, 35868813

[11] Kim Y Lumpkin A Lochbaum M Stegemeier S Kitten K. Promoting physical activity using a wearable activity tracker in college students: a cluster randomized controlled trial. J Sports Sci. (2018) 36:1889–96. doi: 10.1080/02640414.2018.1423886, 29318916

[12] Stecher C Chen C-H Codella J Cloonan S Hendler J. Combining anchoring with financial incentives to increase physical activity: a randomized controlled trial among college students. J Behav Med. (2024) 47:751–69. doi: 10.1007/s10865-024-00492-4, 38704776

[13] Amagai S Pila S Kaat AJ Nowinski CJ Gershon RC. Challenges in participant engagement and retention using mobile health apps: literature review. J Med Internet Res. (2022) 24:e35120. doi: 10.2196/35120, 35471414 PMC9092233

[14] Michie S Richardson M Johnston M Abraham C Francis J Hardeman W . The behavior change technique taxonomy (v1) of 93 hierarchically clustered techniques: building an international consensus for the reporting of behavior change interventions. Ann Behav Med. (2013) 46:81–95. doi: 10.1007/s12160-013-9486-6, 23512568

[15] Hardeman W Houghton J Lane K Jones A Naughton F. A systematic review of just-in-time adaptive interventions (JITAIs) to promote physical activity. Int J Behav Nutr Phys Act. (2019) 16:31. doi: 10.1186/s12966-019-0792-7, 30943983 PMC6448257

[16] Nahum-Shani I Smith SN Spring BJ Collins LM Witkiewitz K Tewari A . Just-in-time adaptive interventions (JITAIs) in mobile health: key components and design principles for ongoing health behavior support. Ann Behav Med. (2018) 52:446–62. doi: 10.1007/s12160-016-9830-8, 27663578 PMC5364076

[17] Sallis JF Saelens BE. Assessment of physical activity by self-report: status, limitations, and future directions. Res Q Exerc Sport. (2000) 71:1–14. doi: 10.1080/02701367.2000.11082780, 25680007

[18] Li X Tan WH Zheng X Dou D Wang Y Yang H. Effects of digital monitoring and immediate feedback on physical activity and fitness in undergraduates. Educ Inf Technol. (2025) 30:3743–69. doi: 10.1007/s10639-024-12990-8

[19] Reis RS Salvo D Ogilvie D Lambert EV Goenka S Brownson RC . Scaling up physical activity interventions worldwide: stepping up to larger and smarter approaches to get people moving. Lancet. (2016) 388:1337–48. doi: 10.1016/S0140-6736(16)30728-0, 27475273 PMC5193005

[20] Koorts H Eakin E Estabrooks P Timperio A Salmon J Bauman A. Implementation and scale up of population physical activity interventions for clinical and community settings: the PRACTIS guide. Int J Behav Nutr Phys Act. (2018) 15:51. doi: 10.1186/s12966-018-0678-0, 29884236 PMC5994105

[21] Glasgow RE Harden SM Gaglio B Rabin B Smith ML Porter GC . RE-AIM planning and evaluation framework: adapting to new science and practice with a 20-year review. Front Public Health. (2019) 7:64. doi: 10.3389/fpubh.2019.00064, 30984733 PMC6450067

[22] Des Jarlais DC Lyles C Crepaz N TREND Group. Improving the reporting quality of nonrandomized evaluations of behavioral and public health interventions: the TREND statement. Am J Public Health. (2004) 94:361–6. doi: 10.2105/AJPH.94.3.361 PMC1448256 14998794

[23] Hoffmann TC Glasziou PP Boutron I Milne R Perera R Moher D . Better reporting of interventions: template for intervention description and replication (TIDieR) checklist and guide. BMJ. (2014) 348:g1687. doi: 10.1136/bmj.g1687, 24609605

[24] Peng S Yuan F Othman AT Zhou X Shen G Liang J. The effectiveness of e-health interventions promoting physical activity and reducing sedentary behavior in college students: a systematic review and meta-analysis of randomized controlled trials. Int J Environ Res Public Health. (2023) 20:318. doi: 10.3390/ijerph20010318, 36612643 PMC9819541

[25] Laranjo L Ding D Heleno B Kocaballi AB Quiroz JC Tong HL . Do smartphone applications and activity trackers increase physical activity in adults? Systematic review, meta-analysis and metaregression. Br J Sports Med. (2021) 55:422–32. doi: 10.1136/bjsports-2020-102892, 33355160

[26] Buja A Lo Bue R Mariotti F Miatton A Zampieri C Leone G. Promotion of physical activity among university students with social media or text messaging: a systematic review. Inquiry. (2024) 61:469580241248131. doi: 10.1177/00469580241248131 PMC11095173 38742671

[27] Nuttall FQ. Body mass index: obesity, BMI, and health: a critical review. Nutr Today. (2015) 50:117–28. doi: 10.1097/NT.0000000000000092, 27340299 PMC4890841

[28] Qian T Walton AE Collins LM Klasnja P Lanza ST Nahum-Shani I . The micro-randomized trial for developing digital interventions: experimental design and data analysis considerations. Psychol Methods. (2022) 27:874–94. doi: 10.1037/met0000283, 35025583 PMC9276848

